# Supplementary material for: Chronic Heat Stress Can Induce Conjugation of a Novel ermB-Containing ICEFZMF, Increasing Resistance to Erythromycin Among Enterococcus Strains in Diverse Intestinal Segments in the Mouse Model
Source: Antibiotics (Basel). 2025 Apr 30;14(5):460. doi: 10.3390/antibiotics14050460 (PMC12108523; doi:10.3390/antibiotics14050460)
Supplement: Supplementary file 1 [file antibiotics-14-00460-s001.zip › antibiotics-3554498-supplementary.pdf]

Figure S1 Expression of heat shock protein mRNA in the gut of mice. Asterisks showed significant differences from the control group using two-way ANOVA with Bonferroni's multiple comparison test (****P<0.0001, ***P<0.001, **P<0.01, *P<0.05 and ns, not significant).

Figure S2 The mRNA abundance of markers in intestinal stress, inflammation and integrity.Asterisks showed significant differences from the control group using two-way ANOVA with Bonferroni's multiple comparison test (****P<0.0001, ***P<0.001, **P<0.01, *P<0.05 and ns, not significant).

Figure S3-Figure S6 The MIC of erythromycin, chloramphenicol, tetracycline, ampicillin, vancomycin, rifampin and ciprofloxacin among strains; Asterisks showed significant differences from the control group using two-way ANOVA with Bonferroni's multiple comparison test (****P<0.0001, ***P<0.001, **P<0.01, *P<0.05 and ns, not significant).

Figure S7 The clonal relatedness of *Enterococcus* strains. A: The clonal relatedness of *Enterococcus* strains isolated in the study. the strains isolated from the HS group was highlight in red. B: The clonal relatedness of stains isolated in the intestinal colonal assays.

Figure S1

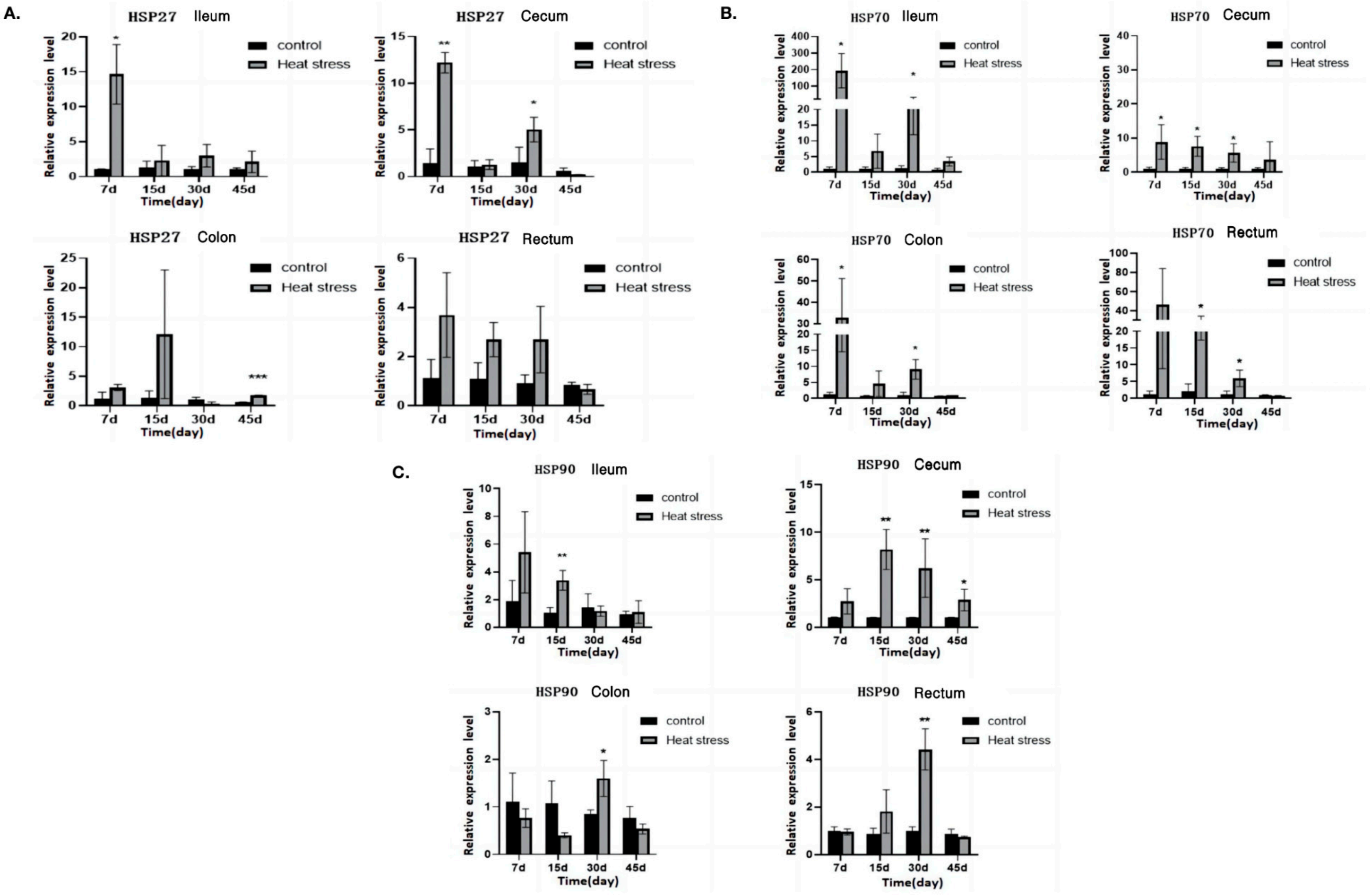
A.
HSP27 Ileum
HSP27 Cecum
HSP27 Colon
HSP27 Rectum
B.
HSP70 Ileum
HSP70 Cecum
HSP70 Colon
HSP70 Rectum
C.
HSP90 Ileum
HSP90 Cecum
HSP90 Colon
HSP90 Rectum
control
Heat stress
Relative expression level
Time(day)
7d
15d
30d
45d

Figure S2

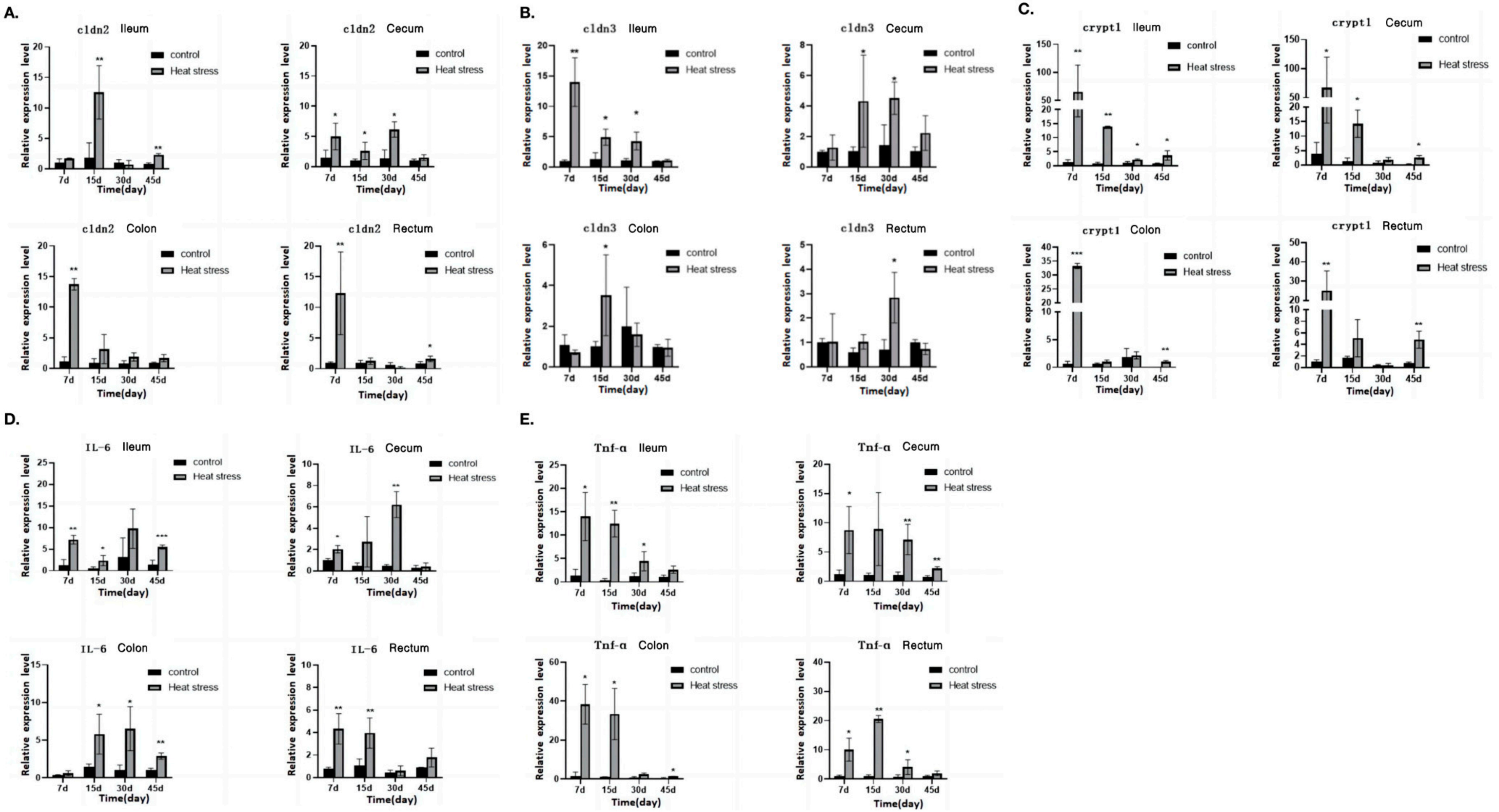

A.
cldn2 Ileum
cldn2 Cecum
cldn2 Colon
cldn2 Rectum
B.
cldn3 Ileum
cldn3 Cecum
cldn3 Colon
cldn3 Rectum
C.
crypt1 Ileum
crypt1 Cecum
crypt1 Colon
crypt1 Rectum
D.
IL-6 Ileum
IL-6 Cecum
IL-6 Colon
IL-6 Rectum
E.
Tnf-α Ileum
Tnf-α Cecum
Tnf-α Colon
Tnf-α Rectum
control
Heat stress
Relative expression level
Time(day)
7d
15d
30d
45d

Figure S3

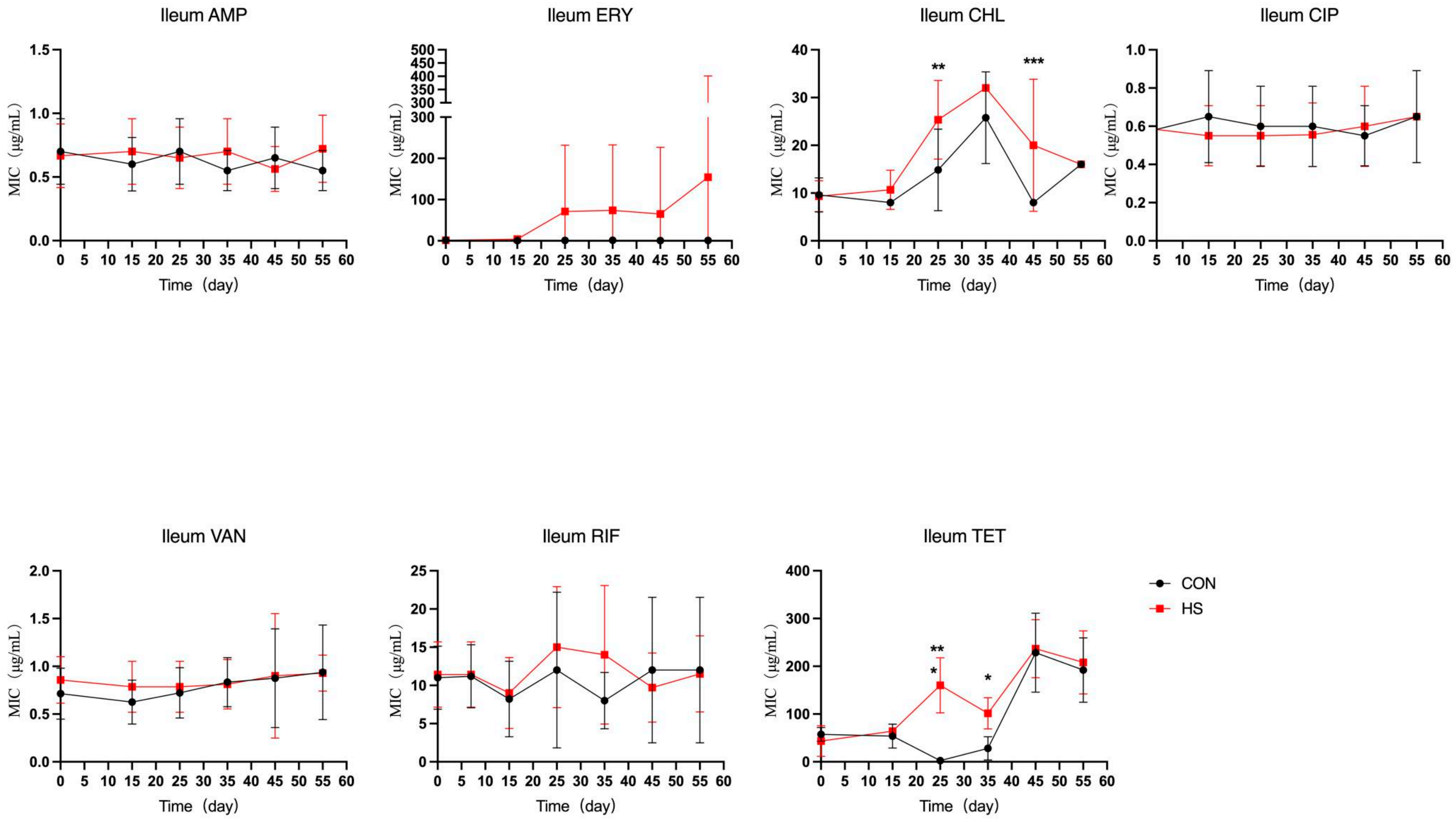
Ileum AMP
Ileum ERY
Ileum CHL
Ileum CIP
Ileum VAN
Ileum RIF
Ileum TET
MIC（μg/mL）
Time（day）
**
***
*
CON
HS

Figure S4

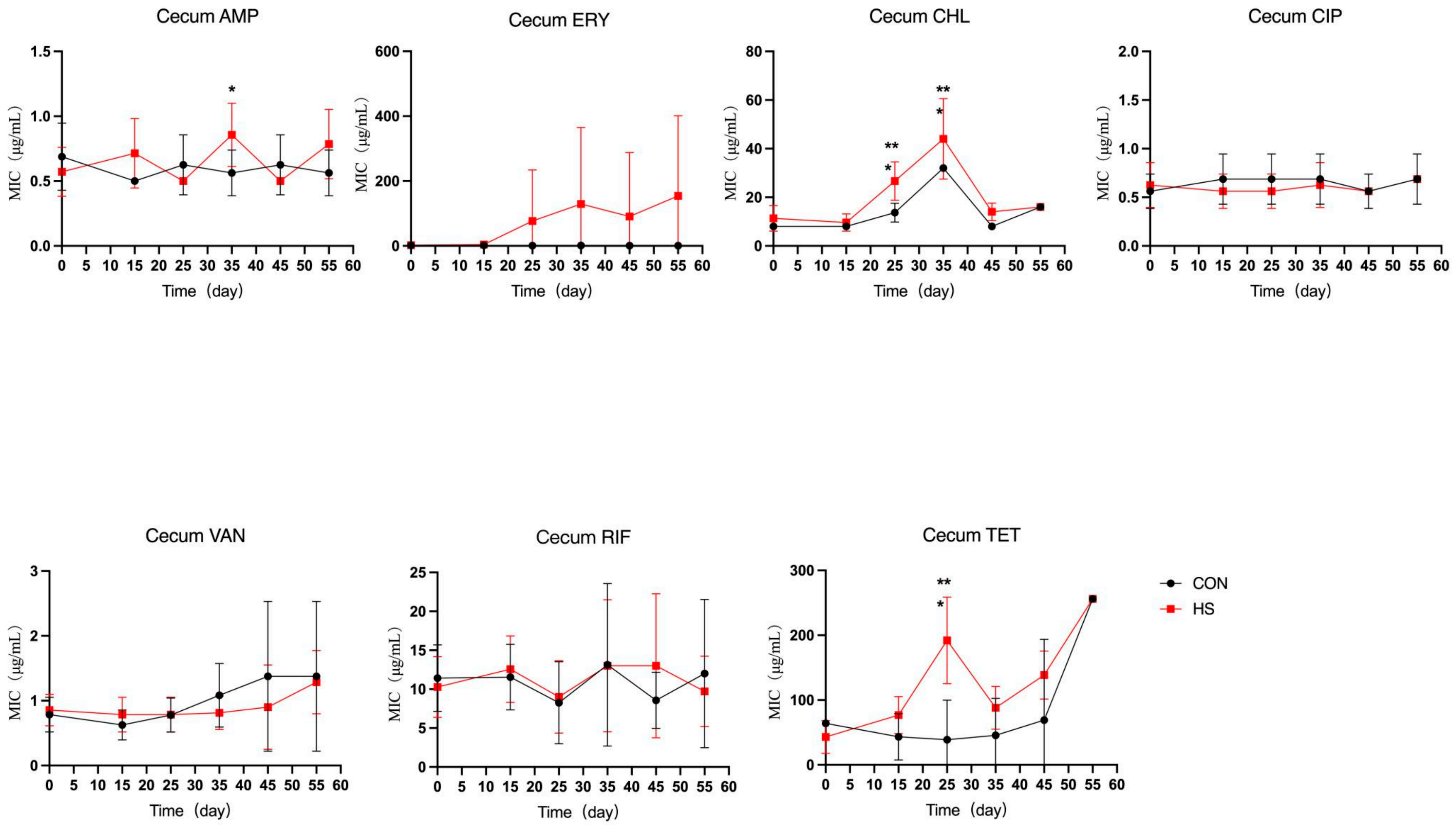
Cecum AMP
Cecum ERY
Cecum CHL
Cecum CIP
Cecum VAN
Cecum RIF
Cecum TET
MIC（μg/mL）
Time（day）
0 5 10 15 20 25 30 35 40 45 50 55 60
*
**
CON
HS

Figure S5

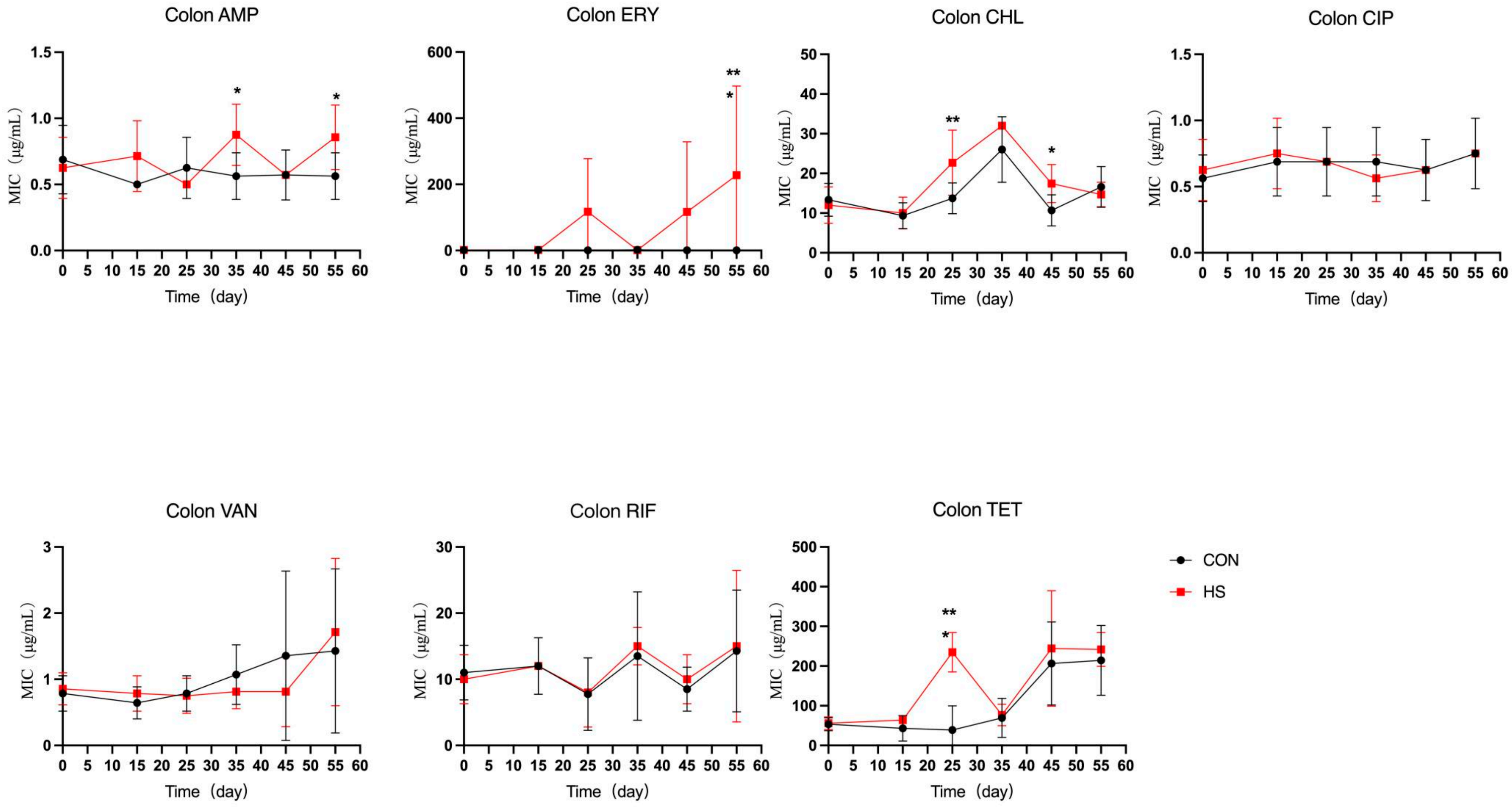
Colon AMP
Colon ERY
Colon CHL
Colon CIP
Colon VAN
Colon RIF
Colon TET
MIC（μg/mL）
Time（day）
CON
HS
*
**

Figure S6

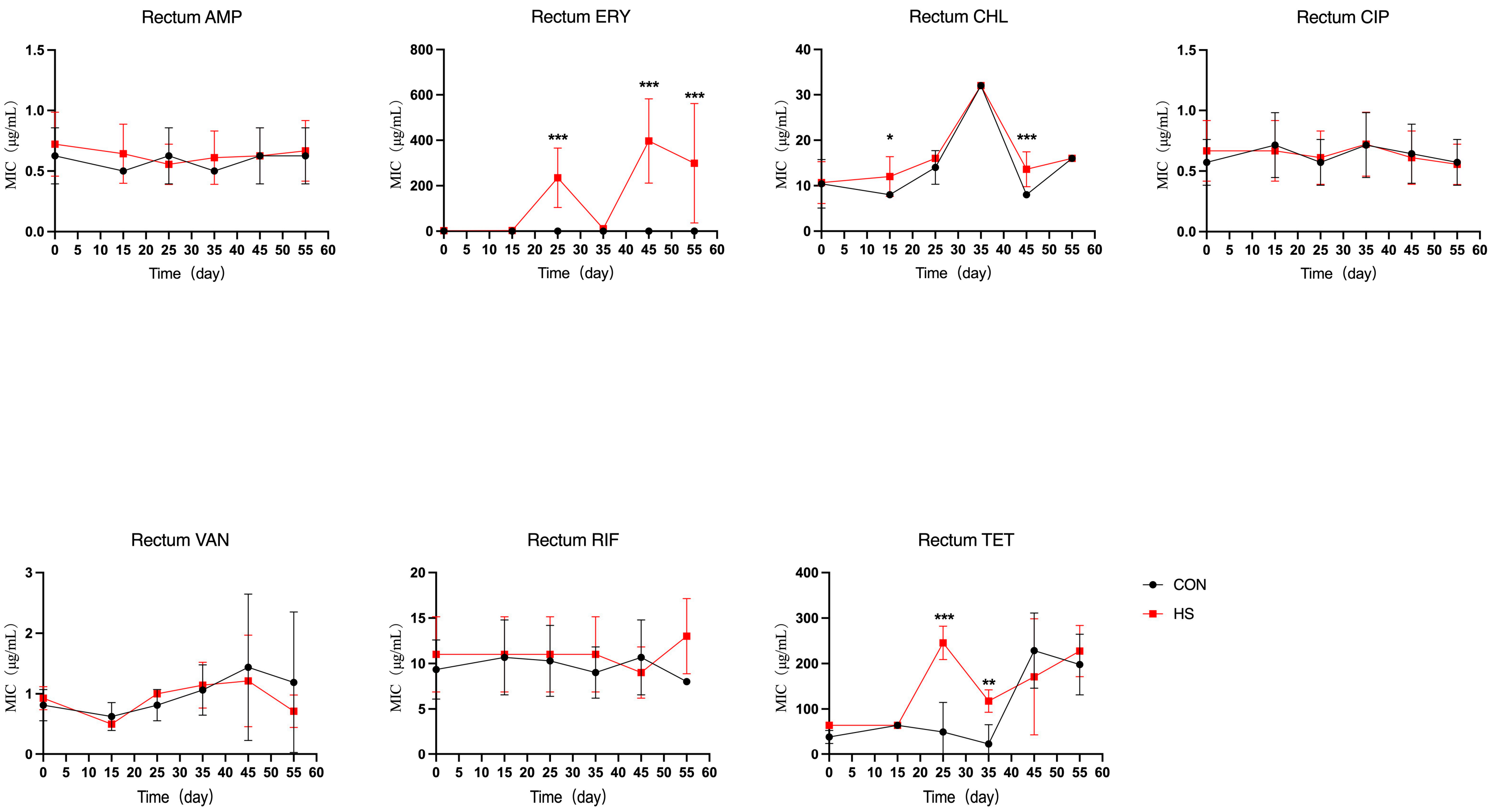
Rectum AMP
Rectum ERY
Rectum CHL
Rectum CIP
Rectum VAN
Rectum RIF
Rectum TET
MIC（μg/mL）
Time（day）
0 5 10 15 20 25 30 35 40 45 50 55 60
*
**
***
CON
HS

Figure S7

A.

B.

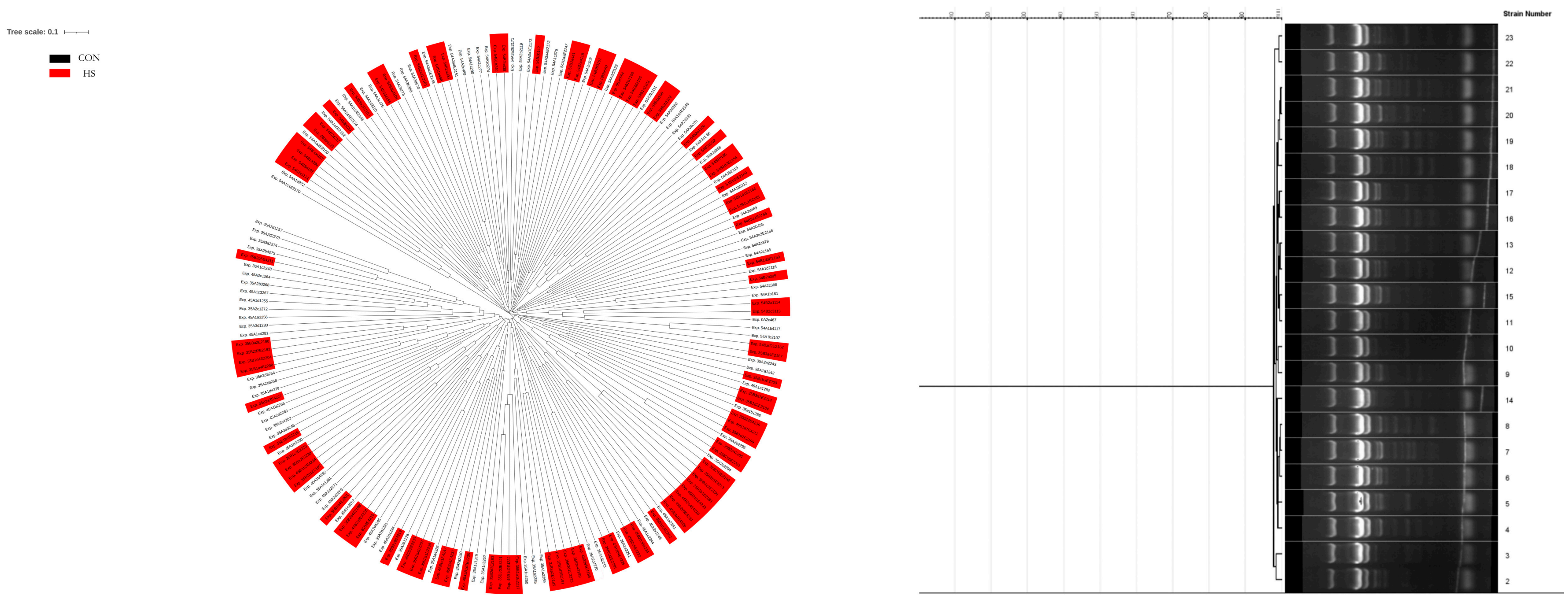

Tree scale: 0.1
CON
HS
Strain Number
23
22
21
20
19
18
17
16
13
12
15
11
10
9
14
8
7
6
5
4
3
2

Table S1 Detection rate of erytromycin resistance bacteria (%)

Days	The CON group				The HS group			
（d）	Ileum	Cecum	Colon	Rectum	Ileum	Cecum	Colon	Rectum
15	0.000%	0.000%	0.000%	0.000%	0.0020%	0.0300%	0.0640%	0.2600%
30	0.000%	0.000%	0.000%	0.000%	0.0050%	0.0061%	0.0025%	0.2400%
45	0.000%	0.000%	0.000%	0.000%	0.0030%	0.0045%	0.0730%	0.0660%
60	0.000%	0.000%	0.000%	0.000%	0.00267%	0.0089%	0.0220%	0.0323%

Table S2 Oligonucleotides used in this study

Primer name	sequence
ERIC-F	5’-ATGTAAGCTCCTGGGGATTCAC-3’
ERIC-R	5’-AAGTAAGTGACTGGGGTGAGCG-3’
β-actin-F	5'-AGATTACTGCTCTGGCTCCTA-3'
β-actin-R	5'-TCGTACTCCTGCTTGCTGAT-3'
HSP27-F	5'-ACTGGCAAGCACGAAGAAAG-3'
HSP27-R	5'-AATGGTGATCTCCGCTGACT-3'
HSP70-F	5'-TCGTGGAGGAGTTCAAGAGG-3'
HSP70-R	5'-TCGTGGAGGAGTTCAAGAGG-3'
HSP90-F	5'-GAACCTCTGCTCAACTGGATG-3'
HSP90-R	5'-CTGGTATGCTTGTGCCTTCA-3'
Cldn2-F	5'- TGAACACGGACCACTGAAAG -3'
Cldn2-R	5'- TTAGCAGGAAGCTGGGTCAG -3'
Cldn3-F	5'- GTGGCCACTGCAGCTACTT -3'
Cldn3-R	5'- GTTTCATGGTTTGCCTGTCTC -3'
Crypt-1-F	5'- TTGGAGACCCCAGAAGGCACTT -3'
Crypt-1-R	5'- CCAGATCTCTCAACGATTCCTCTT -3'
IL-6-F	5'- ACCACGGCCTTCCCTACTTC -3'
IL-6-R	5'- CATTTCCACGATTTCCCAGA -3'
16sRNA-F	5’-AGAGTTTGATCCTGGCTCAG-3’
16sRNA-R	5’-AAGGAGGTGATCCAGCC-3’
TNF-α-F	5'- CGTGGAACTGGCAGAAGAGG -3'
TNF-α-R	5'- AGACAGAAGAGCGTGGTGGC -3'
ermA-F	5'-TGTCCAATTCCACCATCAATAG-3'

ermA-R	5'-AGTCGTGAAGCCTTTTCAGAAT
ermB-F	5'-TCAAGTCTCGATTCAGCAAT-3'
ermB-R	5'-TGTTTACTTTGGCGTGTTTC-3'
mefA-F	5'-AGTATCATTAATCACTAGTGC-3'
mefA-R	5'-TTCTTCTGGTACTAAAAGTGG-3'
fexA-F	5'-TTGGGAAGAATGGTTCAGGG-3'
fexA-R	5'-ATCGGCTCAGTAGCATCACG-3'
optrA-F	5'-AGGTGGTCAGCGAACTAA-3'
optrA-R	5'-ATCAACTGTTCCCATTCA-3'
tet(L)-F	5’-ACTCGTAATGGTGTAGTTGC-3’
tet(L)-R	5’-TGTAACTCCGATGTTTAACACG-3’
tet(S)-F	5’-TGGAACGCCAGAGAGGTATT-3’
tet(S)-R	5’-ACATAGACAAGCCGTTGACC-3’
lsa(A-)F	5’-ATCGGTGAATCAGGCTTAGAGG-3’
lsa(A)-R	5’-GCAGGAAGTGCCGGAGTAGTTT-3’
CE-F	5'-TATTGTCTGGCTTAGCTACCG-3'
CE-R	5'-TAGTACCAATGCAGAAATTCCGAC-3'
IS-F	5'- CTTTCCATACATTTACACCCCTT-3'
IS-R	5'-TTACTTTAACATTTTCCCGTTG-3'
